# Supplementary figures and images for: Genotype networks, innovation, and robustness in sulfur metabolism
Source: BMC Syst Biol. 2011 Mar 7;5:39. doi: 10.1186/1752-0509-5-39 (PMC3060865; doi:10.1186/1752-0509-5-39)

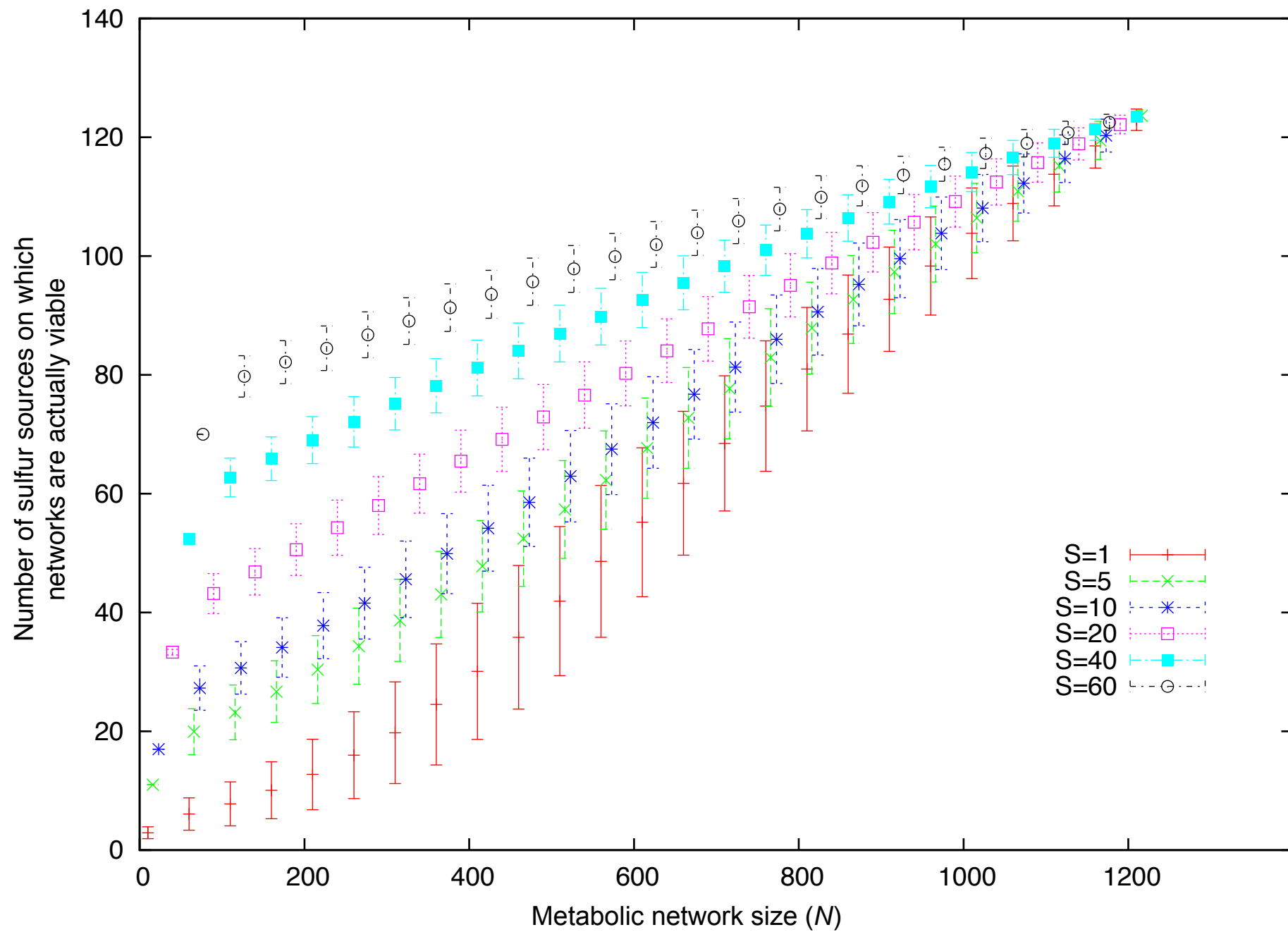

Supplement: Additional file 1 — Random metabolic networks required to be viable on a given number of sulfur sources are generally viable on more sulfur sources. Average number of sulfur sources that random metabolic networks are actually viable in, for varying environmental demands S, and varying metabolic network size N. The figure demonstrates that random metabolic networks required to be viable on a given number S of sulfur sources (as generated by the procedures described in Methods) are generally viable on more than S sulfur sources. Each data point represents an average over 200 random metabolic networks (20 random metabolic networks generated under 10 different sets of environmental demands with the same number S of required sulfur sources). Error bars correspond to one standard deviation. [file 1752-0509-5-39-S1.PDF]

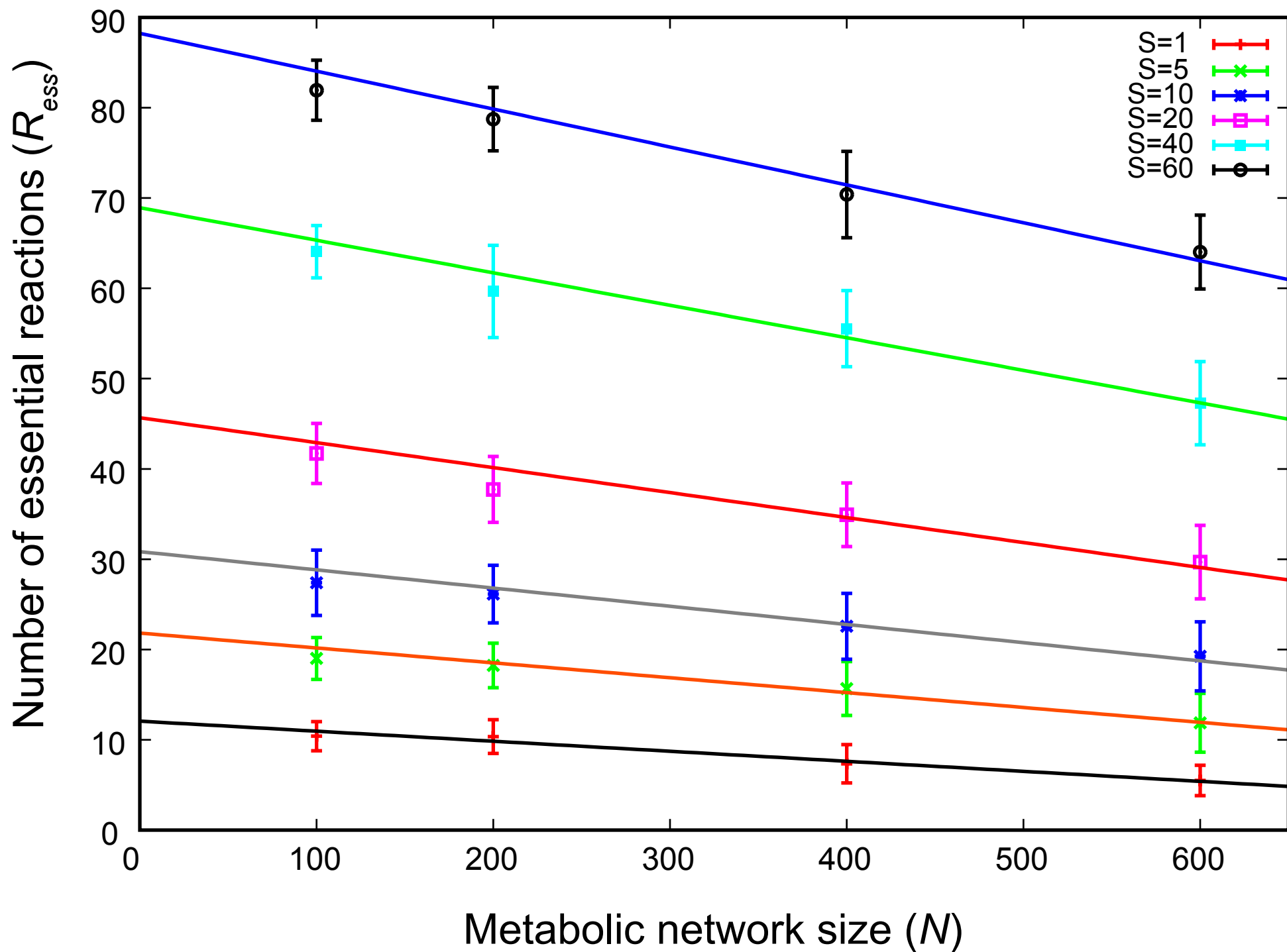

Supplement: Additional file 2 — Number of essential reactions decreases with metabolic network size. Number of essential reactions found in random metabolic networks of different size and for different environmental demands (S). Each data point represents an average over 200 random metabolic networks (20 random metabolic networks generated under 10 different sets of environmental demands with the same number S of required sulfur sources). [file 1752-0509-5-39-S2.PDF]

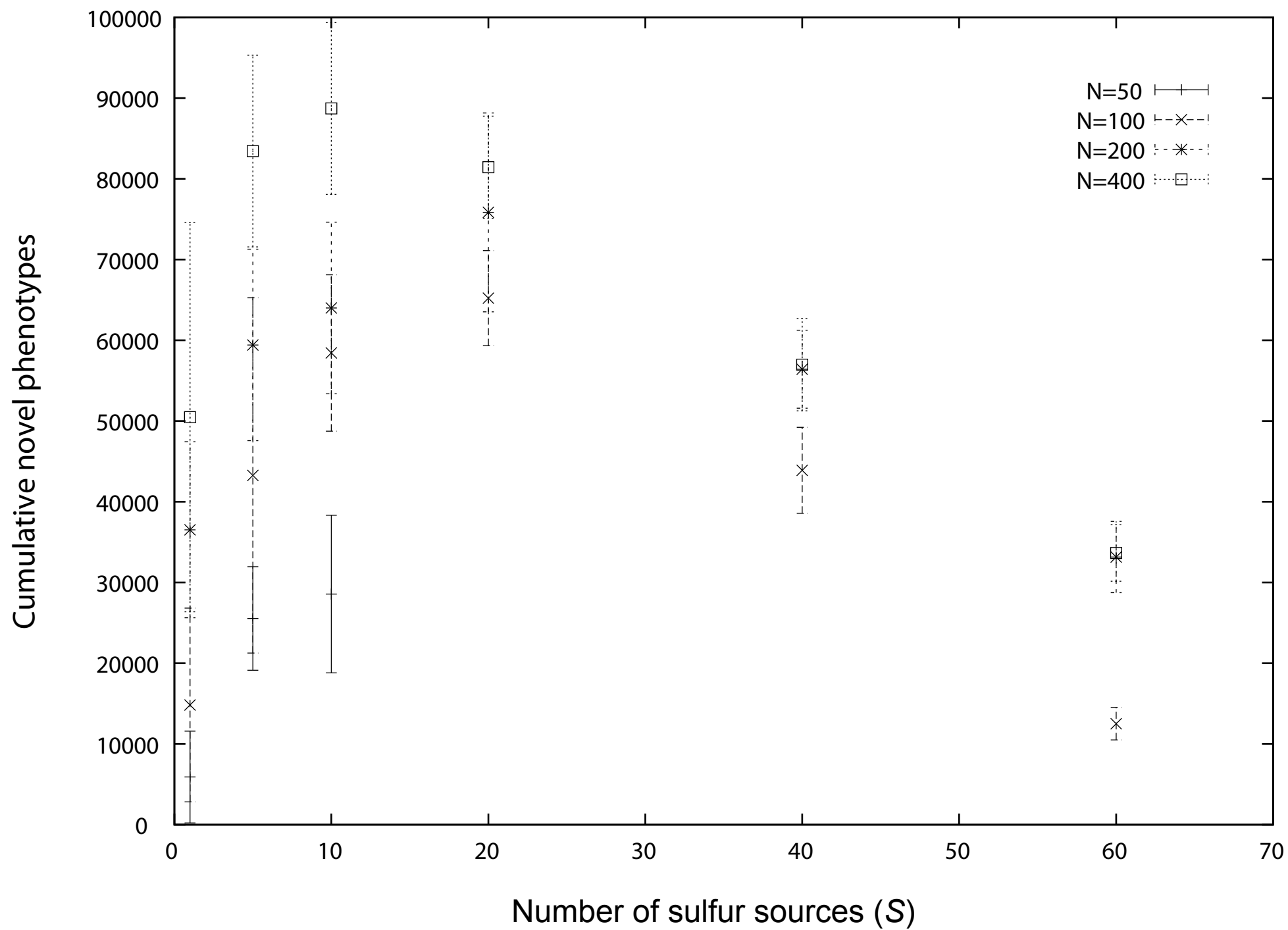

Supplement: Additional file 3 — Cumulative number of novel phenotypes encountered in the neighborhoods of all evolving metabolic networks in a large population. The results are plotted for populations of metabolic networks of different sizes and subject to different environmental demands. Each data point represents an average over 200 simulations, 20 simulations for 10 different sets of environmental demands with the same number S of sulfur sources. Each population consisted of 100 individual metabolic networks. [file 1752-0509-5-39-S3.PDF]

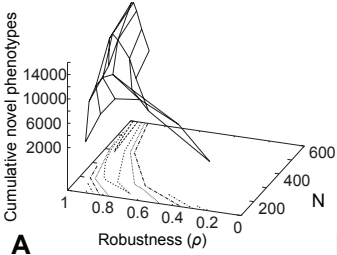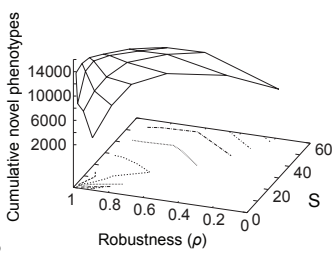

Supplement: Additional file 4 — Number of novel phenotypes as a function of robustness, metabolic network size and environmental demands. Three dimensional plots of number of novel phenotypes found in the (1-mutant) neighborhood of random metabolic networks versus robustness of the metabolic networks and (A) metabolic size N or (B) different environmental demands S. Each data point is an average over 200 metabolic networks (20 random walks for 10 different sets of environmental demands, with the same number S of required sulfur sources). [file 1752-0509-5-39-S4.PDF]

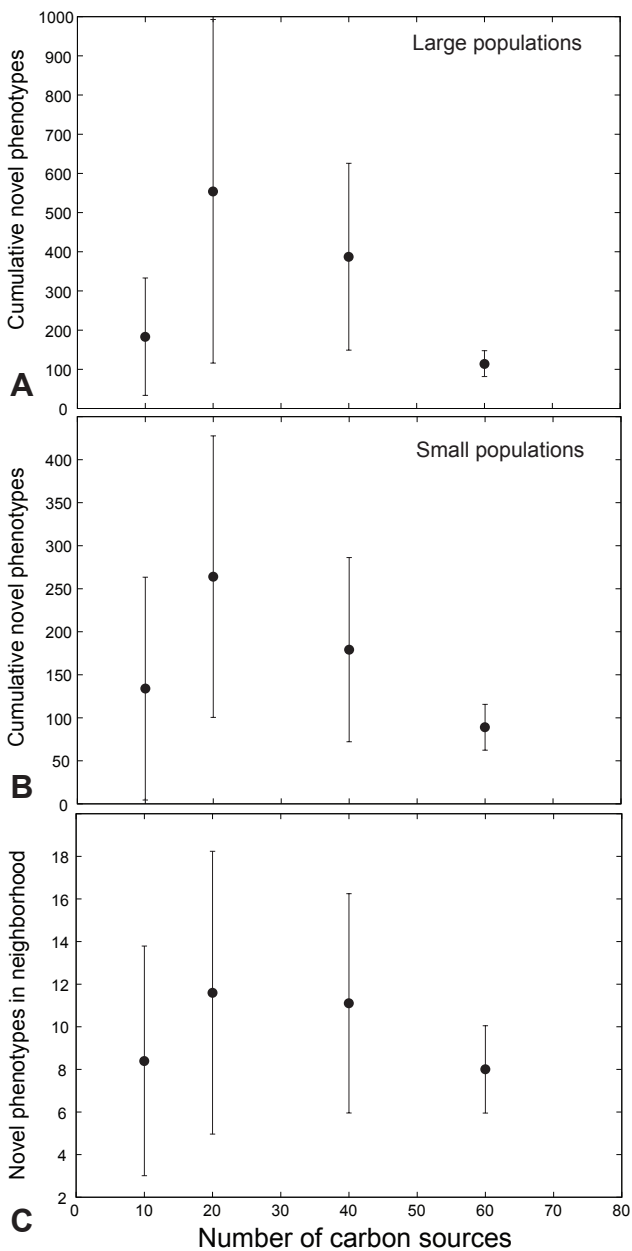

Supplement: Additional file 5 — Cumulative number of novel phenotypes found in the neighborhood of large and small evolving populations of metabolic networks viable in carbon sources. Plot of the cumulative number of novel phenotypes found in the neighborhood of (A) large and (B) small evolving populations of metabolic networks required to be viable in different number of carbon sources. (C) Number of novel carbon utilization phenotypes found in the neighborhood of random metabolic networks. Metabolic networks in these simulations had 931 reactions, the same as the size of the E. coli iJR904 model [5,25]. [file 1752-0509-5-39-S5.PDF]
